# DNA Data Bank of Japan (DDBJ) update report 2022

**DOI:** 10.1093/nar/gkac1083

**Published:** 2022-11-24

**Authors:** Yasuhiro Tanizawa, Takatomo Fujisawa, Yuichi Kodama, Takehide Kosuge, Jun Mashima, Tomoya Tanjo, Yasukazu Nakamura

**Affiliations:** Bioinformation and DDBJ Center, National Institute of Genetics, Mishima, Shizuoka 411-8540, Japan; Bioinformation and DDBJ Center, National Institute of Genetics, Mishima, Shizuoka 411-8540, Japan; Bioinformation and DDBJ Center, National Institute of Genetics, Mishima, Shizuoka 411-8540, Japan; Bioinformation and DDBJ Center, National Institute of Genetics, Mishima, Shizuoka 411-8540, Japan; Bioinformation and DDBJ Center, National Institute of Genetics, Mishima, Shizuoka 411-8540, Japan; Bioinformation and DDBJ Center, National Institute of Genetics, Mishima, Shizuoka 411-8540, Japan; Bioinformation and DDBJ Center, National Institute of Genetics, Mishima, Shizuoka 411-8540, Japan

## Abstract

The Bioinformation and DNA Data Bank of Japan (DDBJ) Center (https://www.ddbj.nig.ac.jp) maintains database archives that cover a wide range of fields in life sciences. As a founding member of the International Nucleotide Sequence Database Collaboration (INSDC), our primary mission is to collect and distribute nucleotide sequence data, as well as their study and sample information, in collaboration with the National Center for Biotechnology Information in the United States and the European Bioinformatics Institute. In addition to INSDC resources, the Center operates databases for functional genomics (GEA: Genomic Expression Archive), metabolomics (MetaboBank), and human genetic and phenotypic data (JGA: Japanese Genotype–Phenotype Archive). These databases are built on the supercomputer of the National Institute of Genetics, whose remaining computational capacity is actively utilized by domestic researchers for large-scale biological data analyses. Here, we report our recent updates and the activities of our services.

## INTRODUCTION

The DNA Data Bank of Japan (DDBJ) is a public database of nucleotide sequences established at the Bioinformation and DDBJ Center (DDBJ Center; https://www.ddbj.nig.ac.jp) of the National Institute of Genetics (NIG) (1). Since 1987, the DDBJ has been accepting annotated nucleotide sequences, issuing accession numbers, and distributing them in collaboration with GenBank at the National Center for Biotechnology Information (NCBI) (2) and the European Nucleotide Archive (ENA) at the European Bioinformatics Institute (EBI) (3). This collaborative framework is known as the International Nucleotide Sequence Database Collaboration (INSDC) (4).

Within this INSDC framework, the DDBJ Center has been maintaining the DDBJ Sequence Read Archive (DRA) for raw sequencing data and alignment information generated by high-throughput sequencing platforms and analysis pipelines (5), the BioProject database for study information and the BioSample database for sample information (1,6). This comprehensive biological data resource enriched with contextual study and sample information is guaranteed free and unrestricted access by the INSDC policy (7). In addition to these INSDC databases, the DDBJ Center maintains the Genomic Expression Archive (GEA) (8) for quantitative data from functional genomics experiments (e.g. gene expression and epigenetics) as a counterpart to the Gene Expression Omnibus at NCBI (9) and the ArrayExpress at EBI (10).

For controlled-access data, the DDBJ Center hosts the Japanese Genotype–phenotype Archive (JGA) to store and distribute human genotype and phenotype data resulting from biomedical research (11,12). JGA is operated in collaboration with the National Bioscience Database Center (NBDC, https://biosciencedbc.jp/en/) at the Japan Science and Technology Agency, in which the NBDC formulates guidelines for sharing human data (https://humandbs.biosciencedbc.jp/en/guidelines) and reviews applications for data submission and access to JGA. JGA also collaborates with the major controlled-access databases, the database of Genotypes and Phenotypes (dbGaP) at NCBI (13) and the European Genome–Phenome Archive (EGA) at EBI (14).

In September 2021, the DDBJ Center renewed the public repository for metabolomics data MetaboBank (https://mb2.ddbj.nig.ac.jp) (1). The new system streamlined the data submission process by adopting a standardized metadata description format.

To improve accessibility and usability, the DDBJ Center has been developing cross-database platforms that can facilitate cooperation between web services and databases. The unified login platform, which was introduced in September 2020, has been incorporated into the DDBJ Fast Annotation and Submission System (DFAST) (15), the newly developed Mass Submission System (MSS) application form (https://mss.ddbj.nig.ac.jp/), and MetaboBank, thereby allowing users to log in with the same user account. In November 2021, the search services for DRA, BioProject and BioSample were integrated into the DDBJ Search (https://ddbj.nig.ac.jp/search), originally developed to index JGA public metadata.

In addition to operating archival databases, the DDBJ Center provides the National Institute of Genetics (NIG) supercomputer as a computational resource for researchers to analyze biological data in Japan. The NIG supercomputer has enhanced its storage system to accommodate the growing demand for data storage for analytical use.

In this article, we report updates to the databases and services of the DDBJ Center. All resources are available at https://www.ddbj.nig.ac.jp and the data are downloadable at ftp://ftp.ddbj.nig.ac.jp and https://ddbj.nig.ac.jp/public/.

## DDBJ ARCHIVAL DATABASES

### Data contents: unrestricted and controlled-access databases

The numbers of annual submissions to the DDBJ Center are summarized in Table 1. In 2021, DDBJ accepted 15 573 submissions for nucleotide sequences, among which 88.5% were contributions from domestic Japanese research groups. This number increased by 128% over the previous year, mostly attributable to the bulk submission of third-party data (TPA) entries for metagenome-assembled genome (MAG) sequences by a group at the University of Tokyo (16). The DDBJ has periodically released all public DDBJ/ENA/GenBank nucleotide sequence data in a flat-file format. The latest periodical release of June 2021 contains 2 750 856 069 sequences and 18 755 444 190 605 bp, and the DDBJ contributed 3.74% of the sequences and 2.08% of the base pairs.

**Table 1.** The Numbers of annual submissions to the DDBJ Center

	2019	2020	2021
*Unrestricted databases*			
Nucleotide sequences	6 688	6 836	15 573
DRA high-throughput sequencing data	1 735	1 967	2 066
GEA functional genomics experiments	61	84	61
*Controlled-access database*			
JGA human genotype and phenotype data	59	72	250

In addition, the DRA accepted 2066 submissions of high-throughput sequencing data in 2021. As of September 2022, the DRA distributed 13 PB of sequencing data in SRA (11.7 PB) and FASTQ (1.3 PB) formats. In 2021, the GEA accepted 61 submissions of data from functional genomics experiments, and 140 experiment datasets were publicly available via the FTP site (ftp://ftp.ddbj.nig.ac.jp/ddbj_database/gea) as of September 2022.

Furthermore, in 2021, the JGA accepted 250 submissions, amounting to 410 TB of data. As of end of 2021, the JGA has distributed 240 studies, 396 471 samples, and 453 TB of human data. Summaries of these studies are available to the public on the DDBJ Search (https://ddbj.nig.ac.jp/search) and the NBDC (https://humandbs.biosciencedbc.jp/en/data-use/all-researches) website. Users are required to submit data usage requests to the NBDC to access the individual-level data from these public studies. In 2021, there were 208 requests. An overview of these statistics is available on our website (https://www.ddbj.nig.ac.jp/statistics/index-e.html).

### MetaboBank

The original MetaboBank was launched in October 2020 as a public repository for metabolomics research (1). To accommodate the other INSDC resources more closely, its data model and submission format were completely redesigned as Version 2 in September 2021. Its metadata are now described in the MicroArray Gene Expression Tabular (MAGE-TAB) format (17) for compatibility with the functional genomics data in GEA and ArrayExpress. This format has also gained popularity in proteomics (18). Another major update is cross-referencing the BioProject and BioSample databases. Recent metabolomics research is often coupled with transcriptomics or other omics information. To associate information across different research data, the existing INSDC framework is the best choice for data integration.

### Open sharing of SARS-CoV-2 genome sequence

Since 2021, the NIG and DDBJ Center have been working on the molecular epidemiological investigation of SARS-CoV-2 in collaboration with Shizuoka Prefecture, where NIG is located. Collaboration with Hamamatsu City, a government-designated city in Shizuoka Prefecture, has also been in progress since April 2022. The annotated genome sequences determined in the collaborations are submitted to the DDBJ as an activity of the Japan COVID-19 Open Data Consortium. As of September 2022, 4422 genome sequences have been made publicly available at INSDC, and the data from Shizuoka Prefecture are also registered at GISAID (https://www.gisaid.org/).

The customized version of DFAST, DFAST_VRL, is available at https://dfast.ddbj.nig.ac.jp/dfv/, which internally uses the Viral Annotation DefineR (VADR) (19) of NCBI to annotate the SARS-CoV-2 genome. DFAST_VRL is also available as a standalone command-line tool (https://github.com/nigyta/dfast_vrl).

## DDBJ SYSTEM UPDATE

### Services for submitting biological data

**Figure 1.**
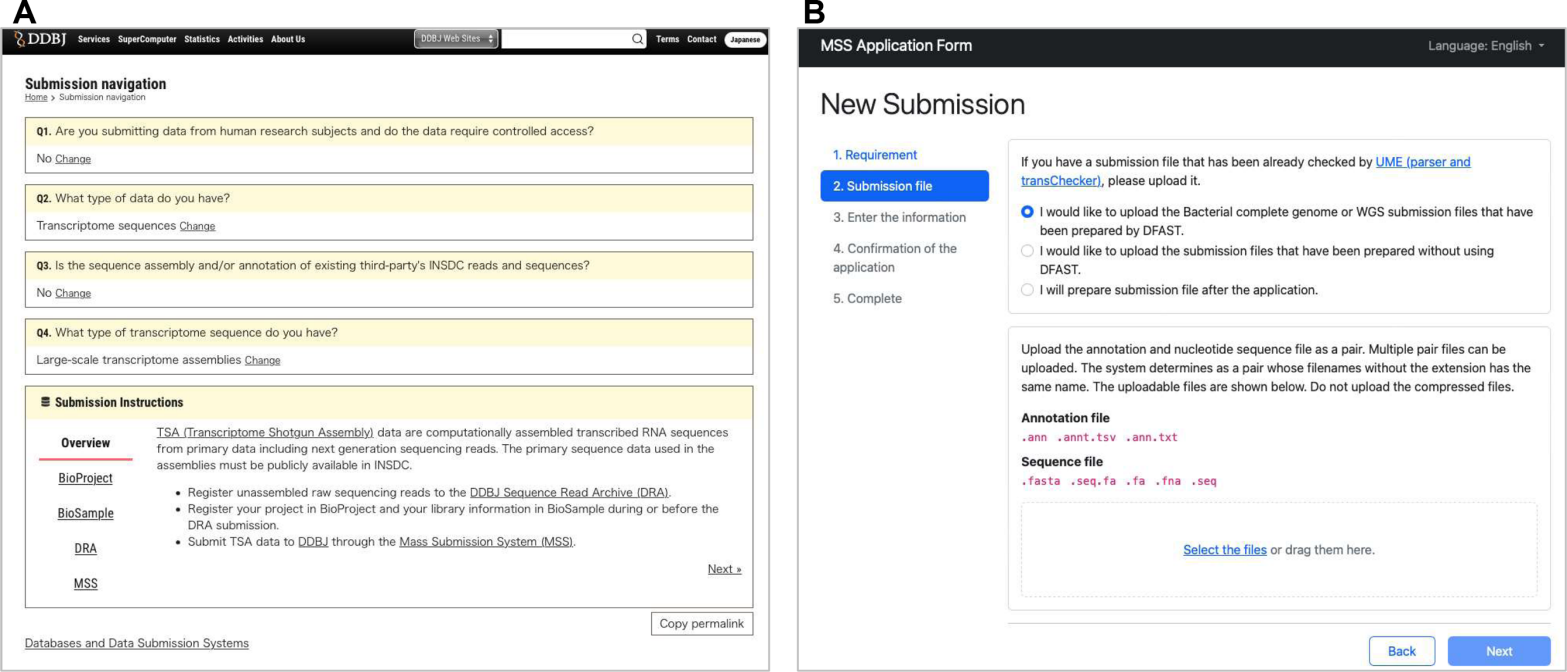
(**A**) The navigation page for data submission. Appropriate databases are suggested depending on the user's data type. (**B**) MSS Application Form. Users can upload data submission files through the interactive user interface.

As the DDBJ Center operates various types of databases, users are apt to be confused about which databases their data should be submitted to. The DDBJ Center released a navigation page (https://www.ddbj.nig.ac.jp/submission-navigation-e.html) for data submission where users can interactively find appropriate databases depending on their data types (Figure 1A).

To simplify the data submission process to DDBJ, the unified login platform, originally introduced in September 2020 for data submission and access to JGA, was incorporated into other services at the DDBJ Center. Users can apply for a new user account at the D-way DDBJ Submission Portal (https://ddbj.nig.ac.jp/D-way/login_form) and can log in to other websites such as DFAST and the MSS Application Form using the same account. DFAST, a genome annotation and data submission pipeline for prokaryotes, provides a job history page to logged-in users, where users can easily browse and manage their annotation jobs. The MSS Application Form (https://mss.ddbj.nig.ac.jp/) was released in June 2022 as a new user interface for relatively large-scale nucleotide data submission to alleviate the communication cost between data submitters and curators in the DDBJ Center (Figure 1B). Users can sign in to the website with a D-way account to upload data submission files. The submitted files are then transferred to the curators together with the information necessary for data validation, which contributes to saving time for the registration of the genome data.

### Services for retrieving biological data

DDBJ Search (https://ddbj.nig.ac.jp/search) was initially launched as an indexing service for JGA’s public metadata (12). As of the update in November 2021, the DDBJ Search was enhanced to include metadata for DRA, BioProject, and BioSamples. It is implemented using ElasticSearch, enabling quick and scalable cross-database search with flexible faceted navigation, and aims to index the metadata of all the databases in the DDBJ Center in future updates. The indexing of metadata related to reference literature is also underway in collaboration with the Database Center for Life Science (DBCLS).

### The NIG supercomputer

The NIG supercomputer system serves as a computational resource for the construction and operation of databases in the DDBJ Center, and is also provided to domestic Japanese researchers for academic purposes in life sciences. The current system, which was installed in March 2019, consists of 243 computational nodes with 15280 CPU cores in total and is equipped with 27.9 PB of sequencing data archiving storage (12.9 PB disk and 15 PB tape) and 16.8 PB of large-scale parallel distributed file systems. Approximately one-third of the computational nodes are allocated to the archival databases operated by the DDBJ Center, the remaining half to the controlled-access section for personal genome analyses, and the rest to the general-purpose analysis section.

As the amount of biological data increases, the need for reproducible analytical platforms also increases. In the NIG supercomputer system, >2000 types of biological software are provided as Apptainer (Singularity) container images, which are obtained from the Biocontainers project (https://biocontainers.pro/) (20). With these containers, users can conduct various types of analyses without investing the time and effort to install the software. Predefined analytical pipelines, such as the DFAST prokaryotic genome annotation pipeline and Rhelixa RNA-seq pipeline, are also available as Apptainer containers. The Apptainer container for the AlphaFold pipeline (https://github.com/deepmind/alphafold) (21), as well as its reference data, are also provided so that it can be run with a GPU on dedicated computational nodes. In addition, the NIG supercomputer can be used as a computational infrastructure for external workflow execution services (WES). The DDBJ WES was developed in collaboration with DBCLS, and its beta version is now available (https://ddbj.nig.ac.jp/wes/). It is based on Sapporo (22), which is an implementation of the Global Alliance for Genomics and Health (GA4GH) WES standard (https://ga4gh.github.io/workflow-execution-service-schemas/docs/) and provides graphical interfaces to execute analytical pipelines described in a workflow language, such as Nextflow, Workflow Description Language, and Common Workflow Language, on the NIG supercomputer. The PortablePipeline (https://github.com/c2997108/OpenPortablePipeline) can also use the NIG supercomputer as a computational engine. This GUI application can perform predefined pipelines on a remote server, including supercomputer systems.

## FUTURE DIRECTION

With the advancement of biological measuring technologies, the diversity and amount of data submitted to the DDBJ Center are rapidly expanding. To accommodate such diverse biological data, DDBJ launched, in addition to conventional nucleotide sequence databases, DRA for archiving next generation sequencing data (2008), JGA for individual-level genetic and phenotypic data (2013), GEA for functional genomics data (2018), and MetaboBank for metabolome data (2020) during the past decade. We are currently developing an archival database for human variation, the Japan Variation Archive (JVar), the data of which will be exchanged with the dbSNP and dbVar of NCBI in the future.

However, these databases are sometimes built upon a different type of data model or user interface, resulting in a complex database system with low usability or maintainability. To alleviate this, we are working towards the integration of database services through the development of an application consisting of microservice units and the standardization of the data model. An example is the unified login system introduced in recent years. It has already been implemented in several web services in the DDBJ Center, not only providing a common interface for user authentication but also enhancing the linkage between web services. Further, we plan to make our data validation pipelines available as open-source software, which will reduce the burden of data validation for both submitters and curators by enabling user-side validation before data submission.

## DATA AVAILABILITY

All resources are available at https://www.ddbj.nig.ac.jp and the data are downloadable at ftp://ftp.ddbj.nig.ac.jp and https://ddbj.nig.ac.jp/public/.
